# Antibody-Based Agents in the Management of Antibiotic-Resistant *Staphylococcus aureus* Diseases

**DOI:** 10.3390/microorganisms6010025

**Published:** 2018-03-13

**Authors:** Pietro Speziale, Simonetta Rindi, Giampiero Pietrocola

**Affiliations:** 1 Department of Molecular Medicine, University of Pavia, 27100 Pavia, Italy; srindi@unipv.it (S.R.); giampiero.pietrocola@unipv.it (G.P.); 2 Department of Industrial and Information Engineering, University of Pavia, 27100 Pavia, Italy

**Keywords:** *Staphylococcus aureus*, virulence factors, antibodies, passive immunization

## Abstract

*Staphylococcus aureus* is a human pathogen that can cause a wide spectrum of diseases, including sepsis, pneumonia, arthritis, and endocarditis. Ineffective treatment of a number of staphylococcal infections with antibiotics is due to the development and spread of antibiotic-resistant strains following decades of antibiotic usage. This has generated renewed interest within the scientific community in alternative therapeutic agents, such as anti-*S. aureus* antibodies. Although the role of antibodies in the management of *S. aureus* diseases is controversial, the success of this pathogen in neutralizing humoral immunity clearly indicates that antibodies offer the host extensive protection. In this review, we report an update on efforts to develop antibody-based agents, particularly monoclonal antibodies, and their therapeutic potential in the passive immunization approach to the treatment and prevention of *S. aureus* infections.

## 1. Introduction

*Staphylococcus aureus* is one of the most common opportunistic human pathogens and the causative agent of diseases ranging from mild infections to severe diseases such as bacteremia, sepsis, pneumonia, endocarditis, and osteomyelitis [1]. *S. aureus* is also the etiological agent of medical device-associated infections, such as central venous catheter sand orthopedic implants, and of surgical wound infections [2]. Antibiotic resistance has dramatically increased the burden of staphylococcal diseases. Indeed, strains resistant to “old” (penicillin and methicillin) and new generation antibiotics (daptomycin and linezolid) were isolated soon after their introduction in the market. This serious drawback and the consideration that antibiotic therapy is not always effective have rekindled interest in the pathophysiology of *S. aureus* and generated new efforts to develop antibody-based agents. Along this line, a large group of *S. aureus* virulence factors has been identified and characterized in recent years. These factors are mostly surface-associated or secreted proteinaceous products.

A significant number of surface proteins act as receptors for extracellular matrix components and play additional roles in biofilm formation and the recruitment of immunomodulators [3]. *S. aureus* has also evolved a series of secreted products that collectively form the secretome. Functionally, secreted proteins/peptides include factors that interfere with complement C3 and C5 convertase activities and reduce the chemotactic activity of neutrophils, an array of lytic enzymes that promote tissue destruction and bacterial spread, super antigens that contribute to septic shock and toxins that damage host tissues.

In this review, we examine the therapeutic potential of these staphylococcal virulence factors as determinants for antibody generation and discuss antibody-mediated protection in the passive immunization approach to *S. aureus* infections.

## 2. Staphylococcus Virulence Factors

### 2.1. Surface Proteins

A large number of surface-expressed proteins of *S. aureus* belong to the families of cell wall-anchored (CWA) proteins and lipoproteins.

The primary reservoir of *S. aureus* in humans is the moist squamous epithelium of the *vestibulum nasi* [4], although some studies have highlighted prevalent colonization by this bacterium of the oropharynx under certain circumstances [5]. Other important habitats are the skin [6], intestine [7], and vagina [8]. From these primary colonization sites of infection, bacteria can be disseminated through the extracellular fluids and spread to deeper host tissues, most notably bone tissue and cardiac valves, which are particularly rich in proteins such as fibrinogen, fibronectin, and collagen.

To colonize these different environments, *S. aureus* expresses a variety of surface adhesins that mediate attachment to the tissues, mostly belonging to the class of CWA. CWA proteins are covalently joined to the peptidoglycan scaffold through a highly conserved Leu-Pro-X-Thr-Gly (LPXTG) motif by the membrane-associated sortase A. Two CWA proteins have been shown to promote bacterial adhesion to squamous cells in vitro: clumping factor B (ClfB) [9] and iron-regulated surface determinant A (IsdA) [10]. ClfB, so named for its ability to bind fibrinogen [11,12], also binds to cytokeratin 10 [13] and loricrin [14], two dominant components of squamous cells. Other CWA proteins involved in bacterial adherence to nares are SdrC, SdrD, and SasG [15,16], as well as the recently discovered SasX [17].

Fibronectin-binding proteins FnBPA and FnBPB and clumping factor A (ClfA) are the most relevant adhesins that bind to fibrinogen [3,18,19,20]. Furthermore, as a result of their fibronectin-binding activity [21,22], FnBPA and FnBPB mediate *S. aureus* colonization of host tissues [23].

Cna, a CWA protein that specifically binds to collagen [24] and laminin [25], has proven to be a virulence factor in several infectious diseases such as endocarditis [26], osteomyelitis [27], keratitits [28], and arthritis [29]. Besides its well-recognized role as an adhesin [30], Cna is a C1q binder and acts as an inhibitor of the classical complement pathway [31]. To further interfere with the action of innate immune cells, *S. aureus* utilizes the cell-wall-anchored and secreted Staphylococcal protein A (SpA). SpA binds to the Fc region of host antibodies and this binding results in the incorrect orientation of the IgG on the surface of the bacterial cells, and impairment of Fc-mediated effector functions. The interaction of SpA with IgM Fab triggers the proliferation and depletion of B cells, resulting in failure of adaptive immune responses [32]. SpA also binds to osteoblasts and generates a variety of coordinated signals that determine bone destruction in osteomyelitis [33,34]. Moreover, SpA induces differentiation of osteoclasts and bone resorption [35]. A structurally and functionally SpA-related protein distributed between cell surface and growth medium is Sbi, a multidomain protein with two immunoglobulin binding domains that associate with the Fcγ-domain of vertebrate IgG [36]. Adenosine synthase A (AdsA) is a unique sortase-anchored enzyme that catalyzes the formation of adenosine via the dephosphorylation of adenosine mono-, di-, and triphosphates. Adenosine causes its effects by binding to G protein-coupled receptors [37] and adenosine/receptor interaction generates signaling cascades that elicit inhibition of platelet aggregation, neutrophil superoxide burst, degranulation, and activation of T cells [38].

*S. aureus*, although considered an extracellular pathogen, can invade a variety of non-professional phagocytic cells such epithelial and endothelial cells, explaining its capacity to colonize mucosa and its persistence in tissues after bacteremia. The underlying major molecular mechanism of invasion of these cells involves FnBPs [39,40,41]. Specifically, *S. aureus* interacts via FnBPs with the extracellular matrix protein fibronectin which in turn binds to α_5_β_1_ integrin on the host cell surface and this is sufficient to induce uptake of bacteria [39,40,42,43]. The ternary complex promotes integrin clustering and a relay of signals that result in cytoskeletal rearrangement, endocytosis, and final bacterial internalization [44]. The iron-regulated surface determinant B (IsdB) protein, the primary role of which is to capture iron from host hemoglobin, also promotes bacterial invasion into human cells [45]. As a consequence of internalization, host cells can be damaged by cytotoxins produced by internalized bacteria (see below) [46]. Notably, internalized bacteria are protected against attack from extracellular host defenses (antibodies) and become virtually resistant to antibiotic treatment.

*S. aureus* expresses about 50 surface-exposed lipoproteins (Lpp), which are involved in different cellular functions. All the Lpps present a common sequence motif, known as the LipoBox, containing an invariant Cys residue that allows the protein to be covalently bound to the membrane through a thioether linkage [47]. Of the total lipoproteins identified so far, three Lpps, the ferric hydroxamate-binding Lpp named FhuD2 (ferric hydroxamate uptake D2), the conserved staphylococcal antigen 1A (Csa1A), and the manganese transporter C (MntC), have been more closely investigated to establish their role in bacterial fitness, virulence and vaccinal potential. Among the surface-associated proteins, a specific mention is deserved by PBP2a, a membrane-associated protein belonging to the family of PBPs (penicillin-binding proteins). PBP2a “catalyzes the crosslinking reaction between two adjacent peptide stems during peptidoglycan biosynthesis” [48]. Following the discovery of the allosteric properties of PBP2a it has been proposed that PBP2a may represent a crucial determinant of resistance to β-lactam antibiotics in methicillin resistant *S. aureus* (MRSA) [48].

### 2.2. Secreted Proteins

*S. aureus* produces a large array of small peptides that target different parts of the host defense system. *S. aureus* secretes a peptide called Staphylococcal Complement Inhibitor (SCIN), which binds to and stabilizes both C42a and C3bBb, thereby reducing the enzymatic activity of the convertases and further C3b formation [49,50]. SCIN blocks phagocytosis and the killing of *S. aureus* cells by the phagocytic cells [51]. The staphylococcal extracellular fibrinogen-binding protein Efb is another secreted protein that induces a conformational change of C3 so that C3 cannot be cleaved to C3b, preventing it from becoming an opsonin [52,53]. Efb can also inhibit the interaction of fibrinogen with neutrophils in vitro, and this property may block neutrophil transmigration and/or fibrin-supported neutrophil activation [54]. Woehl et al. identified the multifunctional extracellular adherence protein (Eap) as a powerful specific inhibitor of both the classical pathway (CP) and lectin pathway (LP). Eap disrupts formation of CP/LP C3 proconvertase (C4bC2) by blocking the binding to C4b of both full-length C2 and its C2b fragment. Consequently, Eap inhibits the deposition of C3b onto the surface of *S. aureus* cells and significantly reduces the level of *S. aureus* opsonophagocytosis and killing by neutrophils [55]. Furthermore, Eap specifically inhibits the activity of neutrophil serine proteases elastase, proteinase 3, and cathepsin, all involved in immune defense [56].

The proteolytic activation of complement results also in the generation of leukocyte chemoattractants C5a and C3a, which trigger leukocyte migration from the blood into the sites of infection. The effects of these chemoattractants on their target cells are mediated by specific membrane-bound receptors. *S. aureus* specifically impairs the response of neutrophils and monocytes to formylated peptides and C5a using a specific secreted peptide. This chemotaxis inhibitory molecule named CHIPS (CHemotaxisInhibitory Protein of *Staphylococcus aureus*) is a small peptide that binds the formyl peptide receptor (FPR) and the C5a receptor (C5aR). Binding of CHIPS to these receptors reduces neutrophil activation and migration to the site of infection [57,58]. Structurally, CHIPS shows homology to the C-terminal domain of staphylococcal superantigen-like (SSL) proteins [59]. SSLs, a large family of staphylococcal superantigen-like exoproteins, interfere with host immune defense by other means. For example, a member of this family, SSL7, affects activation of all three pathways of complement and inhibits the cleavage of C5 to C5a by interfering with its binding to C5 convertases [60,61].

To further contribute to pathogenesis, *S. aureus* secretes the cytolytic toxin Hla and leukocidins. Hla is a toxin that forms heptameric pores in host cell membranes, causing swelling, lysis, and subsequent death of the cells [62]. Hla contributes to *S. aureus* pathogenesis during bacteremia and sepsis, eliciting ADAM10-mediated cleavage of endothelial tight junctions and the consequent alteration of vascular integrity [63]. Hla also targets platelets, preventing repair of the injured endothelial barrier, and causes disfunctions of organs through platelet-neutrophil aggregate formation [64]. Five bi-component leukocidins identified in *S. aureus* show lytic activity toward human phagocytic cells: γhemolysins (HlgAB and HlgCB), Panton–Valentine leukocidin (PVL or LukSF), LukED, and LukGH (also known as LukAB) [65,66]. The two monomers of each leukotoxin, termed S- and F-subunits, assemble to form pores in the membranes of the target cells. Enterotoxins are a family of over 20 secreted toxins with clear superantigenic activity. The enterotoxin with the most powerful superantigenic activity is the staphylococcal enterotoxin B (SEB) [67,68]. SEB interconnects the MHC class II molecules on antigen-presenting cells and Vβ chains of T-cell receptors on lymphocytes, causing an exaggerated immune response, failure of the circulatory system and a potentially fatal condition known as toxic shock syndrome [69,70,71].

## 3. Antibodies in Bacterial Infections

Vaccines and antibodies are the most important alternatives to the use of antibiotics. Unlike vaccination, which induces a specific immune response and protection of individuals against later exposure to the same pathogen, passive immunization is short-term immunity in which preformed exogenous antibodies are transferred to a recipient by natural or artificial transfer of antibodies from an immune individual to a non-immune one. Vaccines need quite some time to become effective and are therefore suitable for preventing infections associated with cardio-thoracic and orthopedic surgery or prolonged implantation of catheters. Conversely, antibodies, once administered, provide immediate protection to individuals who have been exposed to an infectious organism and could represent an effective approach to combat infections associated with emergency interventions. To this end, it is worth mentioning a study in which variable anti-Hla antibody levels in different patient populations (from infants to adults) were observed [72]. Likewise, it was observed by Verkaik et al. that levels of antibodies to toxic shock syndrome toxin-1 (TSST-1), staphylococcal enterotoxin A (SEA), ClfA, and ClfB are higher in persistent *S. aureus* carriers than in noncarriers [73]. While these findings suggest a limitation to a general, potential approach to the use of antibody-based therapy, it could also provide assistance with the identification of subjects that could benefit from the use of therapeutic antibodies (or vaccination).

Antibodies can participate in host defense in many ways: (i) they can neutralize the action of bacterial toxins by binding to them and thereby limiting their binding to the surface of host cells; (ii) antibodies coating a bacterium render it recognizable as foreign by macrophages and neutrophils, which then ingest and destroy it (opsonization); (iii) antibodies form a complex with the surface of certain pathogens and the complex antibody-pathogen directly triggers the activation of the classical pathway and the consequent formation of C3b, which binds covalently to the bacterial surface and promotes pathogen opsonization by phagocytes.

### 3.1. Polyclonal Antibodies to S. aureus in Passive Immunization Therapy

Before antibiotics, serum therapy was the first line of antimicrobial defense system to treat infections [74]. The modern version of serum therapy includes immunoglobulin preparations made of highly purified products from which impurities and virus have been removed, and is prevalently used to treat toxin- and viral-mediated diseases. Polyclonal IgG is also used to prevent mother-to-child transmission of HBV and varicella-zoster virus.

However, when using animal/human serum to treat sick patients, antibodies induce an immune response with low specificity to the infective agent. An immune serum contains such a complex and heterogeneous mixture of molecules that active immunoglobulins would not reach sufficient concentrations at the target site to effectively combat the microbes. Altastaph, an IgG obtained from the plasma of human donors immunized with *S. aureus* type 5 plus type 8 capsule polysaccharide, was the first antibody-based *S. aureus* therapy to go into Phase II clinical trial. Altastaph used in the treatment of bacteremia provided protection in mice infected with *S. aureus* and enhanced bacterial opsonophagocytosis but failed to protect against the development of bacteremia [75,76]. More recently, pooled human sera exhibiting high antibody titers to the staphylococcal fibrinogen-binding proteins ClfA of *S. aureus* and SdrG of *S. epidermidis* (Veronate) were tested as potential therapeutic agents to prevent infections in neonates. Veronate showed an opsonic ability to promote staphylococcal uptake and protection in mice challenged with *S. aureus*, but failed to reduce the incidence of late-onset sepsis in a Phase III clinical trial [77,78].

Passive transfer of purified anti-IsdA or -IsdB antibodies protected naïve mice against abscess formation and lethal intravenous challenge, suggesting that IsdA and IsdB antibodies may provide protection by interfering with the pathogen’s heme–iron scavenging mechanism [79]. Immunotherapy targeting adenosine synthase also induced a decreased severity of *S. aureus* infection in mice infected with *S. aureus*. No information is currently available on the potential effects of AdsA-based immunotherapy in clinical trials [80]. Results obtained by Naghshbandi et al. showed that passive immunization with rabbit purified polyclonal antibodies against recombinant PBP2a results in a significant increase in the survival rate of mice challenged with a lethal dose of MRSA and reduction of bacterial spread to the internal organs, confirming the potential importance of passive immunization with anti-PBP2a antibodies in controlling MRSA infections [81]. Monoclonal antibodies against PBP2a have also been generated and extensively characterized, but their efficacy in immunoprophylaxisis is still to be determined [82,83].

### 3.2. Anti-S. aureus Monoclonal Antibodies in Passive Immunization Approach

Advancement in the discovery and production of monoclonal antibodies (mAbs) significantly contributed to the avoidance of the heterologous sera. The rush to the production of monoclonal antibodies started when Kohler & Milstein discovered the hybridoma technology for the generation of mAbs by mouse cell cultures [84]. Since this first step, molecular cloning and recombinant expression methods have provided tools for “reshaping” the antibody molecule to obtain antibody fragments, humanized or fully human antibodies. A third wave of advancement in the field is currently under development, with subtle antibody engineering enabling the manipulation of the antibody molecule so that it can meet specific therapeutic requirements.

There are many advantages in the use of mAb therapy: (i) first, mAbs in contrast to polyclonal antibodies are chemically defined reagents and exhibit low variability; (ii) the high specificity of mAbs results in little cross-reactivity with both host cells and normal bacterial flora; (iii) mAbs usually target specific microbial epitopes that are important in pathogenesis and this increases the potency of the activity per mass of mAb used [85,86].

MAbs against surface components of *S. aureus*—The mouse mAb Aurexis and its humanized version, tefibazumab, which target ClfA, when used in combination with conventional antibiotics, resulted effective in animal models but exhibited a limited action in a Phase II clinical trial [87,88,89]. Likewise, pagibaximab, a monoclonal antibody which targets lipoteichoic acid (LTA), an important key component of the bacterial Gram-positive cell wall, failed in a phase III sepsis trial [90,91]. The single chain mAb fragment Aurograb, which targets the *S. aureus* ATP-binding cassette (ABC) transporter GrfA, in combination with vancomycin, was demonstrated to be synergistically effective in treating *S. aureus* infection in mice but ineffective in a Phase II trial [92,93,94].

Additional monoclonal antibodies against different antigens have been developed or are being developed at research level. Early production and characterization of anti-*S. aureus* mAbs against the collagen/laminin binding adhesion Cna paved the way for the generation of a variety of mAbs against CWA proteins of *S. aureus*. All the members of the anti-Cna antibody family appeared to recognize conformational epitopes and were able to inhibit the binding of collagen/laminin to *S. aureus* cells [95]. They also blocked attachment of bacteria to collagen/laminin substrates. Notably, some of these mAbs could effectively displace bacteria that had previously adhered to collagen [95]. Schaffer et al. generated an anti-ClfB monoclonal antibody that blocks attachment of *S. aureus* to immobilized cytokeratin 10 and showed that administration of this antibody to mice prior to inoculation with bacteria significantly reduces nasal colonization compared with the colonization observed after passive immunization with an isotype-matched non-inhibitory antibody [9]. From a collection of murine mAbs raised against iron-regulated surface determinant B (IsdB), antibodies that exhibited opsonophagocytic killing of *S. aureus* cells and showed protection in lethal challenge models and a sublethal indwelling catheter model were selected [96].

Anderson et al. generated mAbs to MntC that can bind to *S. aureus* and *S. epidermidis* cells and can be protective in an infant rat immunoprophylaxis model [97]. MAbs against a non-toxigenic mutated isoform of protein A were isolated by Kim et al. and shown to prevent the association of immunoglobulin to protein A and to neutralize the Fc- and Fab-binding activities of SpA. Anti-SpA mAbs also promoted opsonophagocytic killing of MRSA in human and mouse blood, provided protection from abscess formation and stimulated pathogen-specific immune responses in a mouse model of staphylococcal disease [98]. The anti-protein A mAb 3F6 protects neonatal mice against *S. aureus* sepsis and raises protective immunity against subsequent staphylococcal infection [99]. Recently, Varshney et al. identified and isolated several anti-SpA antibodies from healthy human donors and produced these antibodies in mammalian cells in an IgG3 format. One of these antibodies, named 514G3, binds with high affinity SpA via its CDRs, has downstream effector functional and protects mice against lethal challenge with MRSA in a bacteremia model. Importantly, at sub-efficacious doses, 514G3 was able to successfully rescue animals when tested in combination with sub-inhibitory doses of vancomycin [100]. Finally, a mAb named 2H7 raised against the large *Staphylococcus aureus* surface protein A (SasA) should be mentioned. Passive administration of 2H7 induced better survival of mice challenged with *S. aureus* strains and enhanced bacterial clearance in kidneys in both sepsis and peritoneal infection. Prophylactic use of 2H7 also significantly reduced the formation of intraperitoneal abscess in mice with peritoneal infection [101].

### 3.3. MAbs to Secreted Staphylococcal Proteins

A number of mAbs that target secreted virulence factors and their therapeutic validation in animals are under development. Medimmune developed a human monoclonal antibody specific for α-hemolysin (Hla), termed MEDI4893, which binds and neutralizes the Hla domain involved in receptor binding and affords protection to mice challenged with *S. aureus* in a model of acute pneumonia [102,103]. Likewise, Arsanis developed two mAbs (ASN-100), one (ASN-1) showing cross-reactivity against Hla and four other toxins (HlgAB, HlgCB, LukED, & LukSF) and a second one (ASN-2) binding leucocidin LukAB [104,105]. A single cross-reactive antibody with such properties prevented lysis of phagocytes, epithelial cells, and erythrocytes caused by Hla and leukocidins in vitro, and induced significant protection in murine models of pneumonia and sepsis [104].

Therapeutic treatment with humanized anti-SEB antibodies protected mice in a SEB intoxication model as well as in sepsis and deep-tissue infection models [106]. Along this line, administration of human-mouse chimeric high-affinity anti-SEB antibodies attenuated the systemic inflammatory response caused by SEB-producing *S. aureus* and protected from lethal pneumonia [107]. Likewise, passive administration of a combination of mAb-4G3 and mAb-5G4, two mAbs recognizing different epitopes on staphylococcal enterotoxin K (SEK), significantly enhanced survival of mice in a model of SEK-induced lethal shock and sepsis [108].

In a study performed by Rukkawattanakul et al., human monoclonal single chain antibodies (scFvs) were generated that bind to critical amino acid residues of TSST-1. TSST-1 causes proliferation of peripheral blood T lymphocytes and release of huge amounts of pro-inflammatory cytokines resulting in a life-threatening disease known as toxic shock syndrome (TSS). Binding of scFvs to TSST-1 inhibited activation and proliferation of T cells and secretion of pro-inflammatory cytokines, suggesting their potential therapeutic application against TSS [109]. In confirmation of the value and possible use of anti-TSST-1 antibodies as therapeutic agent, Kansal et al. also demonstrated that high levels of anti-TSST-1 antibodies significantly protect women against menstrual toxic shock syndrome [110].

### 3.4. Cocktails of mAbs Targeting Multiple Determinants and Their Efficacy against Staphylococcal Infections

Until now, passive immunization with mAbs or sera targeting single antigens has been successful in animal models of infection but did not protect human patients in clinical trials. To explain this drawback, it has been proposed that immunization with a single surface antigen may not have been sufficient to confer protection, due to the large plethora of redundant virulence factors expressed by *S. aureus*. Hence, Pozzi et al. conjugated *S. aureus* surface polysaccharide PNAG CP5 and CP8 to carrier protein Hla, ClfB, or IsdB, respectively, and separately used these formulations of antigens to immunize rabbits. Each combination showed a high titer of polysaccharide-specific opsonic antibodies and protein specific functional antibodies. In a passive immunization approach PNAG-Hla, CP5-ClfB, and CP8-IsdB bivalent antibody combinations reduced experimental *S. aureus* skin infection, pneumonia, and nasal colonization [111].

A combination of mAbs targeting Hla (MEDI4893) and ClfA (10H10), when tested in animal models of infection, enhanced strain coverage in a mouse bacteremia model and improved survival of the animals compared to that obtained with the individual mAbs. This mAb combination also reduced disease severity in murine dermonecrosis and pneumonia models [112]. Tkaczyy et al. also constructed engineered bispecific antibodies (BISAbs) comprised of an anti-ClfA mAb as scaffold and an anti-Hla single-chain variable fragment (scFv) grafted to the N- or C-terminus of ClfA heavy chains, and tested them for efficacy both in vitro and in vivo. These multimechanistic monoclonal antibodies showed in vitro activity and efficacy comparable to those of the parental mAbs and proved not to be beneficial over the mAb combination in an *S. aureus* bacteremia model, suggesting that mAb combination targeting ClfA and Hla is more promising for future clinical development than the corresponding BISAbs [113].

Recently, Wang et al. developed a hematogenous orthopedic infection model and identified Hla and ClfA as preeminent factors promoting biofilm formation. Neutralizing human mAbs against Hla and ClfA, particularly when used in combination, blocked biofilm formation in vitro and significantly reduced the tendency towards hematogenous orthopedic implant infection and biofilm formation in the implants [114]. As reported in Section 3.3, Arsanis researchers also isolated cross-reactive mAbs with high affinity for Hla and 4 different bi-component leukocidins (HlgAb, HlgCB, LukED, and LukSF) [104].

### 3.5. Antibody–Antibiotic Conjugates

*S. aureus* can be internalized by both phagocytic and non-phagocytic cells. Phagocytic cells opsonize and internalize bacteria through opsonophagocytosis. For non-phagocytic cells, such epithelial or endothelial cells, entry of bacteria can mainly occur through a mechanism involving staphylococcal adhesions FnBPs, fibronectin, and host cell α_5_β_1_ integrin [39,40,41,43]. Survival of *S. aureus* internalized by host cells can not only facilitate the dissemination of the infection via the bloodstream [115], but also contribute to the persistence of infection [116,117]. In fact, most antibiotics are not effective, both in vitro and in vivo, at killing intracellular *S. aureus* due to their poor ability to penetrate the host cells. Recently, a drug composed of a specific antibody targeting a surface component of the wall teichoic acid of *S. aureus* and a covalently conjugated potent antibiotic belonging to the rifamycin class, when used to opsonize bacteria, was shown to efficiently eradicate intracellular cells of *S. aureus* sequestered inside murine macrophages. Parallel studies performed in vivo have shown that this therapeutic platform efficiently reduces the bacterial load in the kidneys, heart, and bone [118].

### 3.6. Lysibodies

Due to their abundance and high conservation, carbohydrates, the major components of the Gram-positive bacterial cell wall, are promising targets for the development of therapeutic antibodies. However, they promote a weak immune response because they induce antibody production by B lymphocytes without the involvement of T lymphocytes. To circumvent these difficulties, Raz et al. constructed a recombinant conjugate made of domains of bacteriophage lysins or staphylococcal autolysins that bind specifically and with high affinity cell wall carbohydrate determinants on the staphylococcal surface in fusion with the Fc region of human IgG. This resulted in the generation of effective therapeutic antibodies named lysibodies. Lysibodies induce *S. aureus* phagocytosis by neutrophils and macrophages and promote fixation of complement on the surface of *S. aureus* cells. Additionally, they protect mice from MRSA in a kidney abscess model of infection and bacteremia model of infection [119].

## 4. Discussion and Perspectives

In this review we have described the variety of virulence factors expressed by *S. aureus* and highlighted the importance of these factors as antigens for the development of therapeutic polyclonal and monoclonal antibodies against *S. aureus* infections. Furthermore, we report the outcome of treatment of infective diseases in humans and animals with antibody-based strategies. Many past and recent reviews have discussed the reasons underlying the success and the failure of these agents as antimicrobials (see, for example Reference [120] and the literature therein). A primary reason for the failure of antibody-based biologic agents in passive immunization is related to the use of antibodies targeting a single cell surface-associated antigen. Secondly, the impact of the immunoglobulin-binding proteins SpA and Sbi that sequester IgGs and impair their function in immune defense mechanisms (phagocytosis and complement activation) have not been considered. Third, poor consideration has been attributed to the expression of several superantigens produced by *S. aureus*, which deeply interfere with normal host immune functions. Moreover, it has not duly considered the non-obvious direct extrapolation of the protection data obtained by treating *S. aureus*-challenged animals (mouse, rabbit, and even non-human primates) with therapeutic antibodies to clinical studies in humans [121]. Lastly, many *S. aureus* clinical isolates present extensive variability in their repertoire of genes encoding virulence determinants such as surface proteins involved in adherence/colonization of the host tissues and soluble factors implicated in immune evasion [122,123]. Consequently, this could impact the efficacy of antibodies against variable antigens. For example, if the amino acid sequence of ClfA is 86% conserved among the strains, the remaining 14% difference in amino acid composition alters ClfA immunogenicity and affects the affinity of anti-ClfA antibodies for the protein variants expressed by *S. aureus* trains [124]. Individual clonal differences are also brought about by insertions of mobile DNA elements, prophages and plasmids, that encode for some of the above discussed virulence factors such as CHIPS and SCIN [125,126].

On the other hand, the prophylactic use of anti-*S. aureus* mAbs may offer better opportunities for the prevention and treatment of *S. aureus* diseases for which antibiotic therapy has not proven effective. These include mAbs that block virulence factors such CHIPS and SCIN and that can consequently restore and revitalize host immunity. In this context, a human monoclonal antibody, named 6D4, that blocks the complement inhibitory activity of SCIN has recently been generated [127]. Of note, the immunodominant *S. aureus* antigen A (IsaA), an invariant, widely expressed cell wall-associated protein, suggested to be involved in growth, division, separation, and survival of *S. aureus* cells [128], may be another promising target for passive immunization in human population. In fact, an IsaA human monoclonal antibody recognizing the N-terminal domain of the protein [129], named 1D9, has been shown to protect mice against bacteremia [130].

Therapeutic mAbs are also in production or under development that are tailored to target the extracellular and intracellular lifestyles of the bacterium. For example, monoclonal antibodies with the ability to block SpA and other superantigens may not only be useful for the general therapy of *S. aureus* diseases in humans but, due to their neutralizing superantigen activity, may also facilitate the development of protective immune responses to many different staphylococcal antigens. Furthermore, it has been proposed that the development of antibody–antibiotic conjugates may be promising both to combat internalized bacteria and bacterial biofilm formation. In this last approach, the antibody could help to recognize a specific antigen expressed by an *S. aureus* biofilm and concentrate and locally release the antibiotic onto the biofilm [131].

Although it has over-emphasized the role of antibodies in the opsonophagocytic killing of *S. aureus* cells and under-appreciated the ability of *S. aureus* to survive within the phagocytic cells, opsonophagocytosis remains an important step to neutralize *S. aureus* infection. The development of the “lysibody” concept may become an important step in the cooptation of poorly immunogenic carbohydrate epitopes and the generation of anti-capsular polysaccharide antibodies promoting phagocytosis of bacteria by macrophages and neutrophils.

Given the complexity and redundancy of virulence factors expressed by *S. aureus*, it is now clear that passive immunization strategies should be based on the use of cocktails of blocking antibodies targeting several antigens and, in the reasonably near future, selection and experimentation of new combinations of mAbs in animals and humans will become the focus of further investigation.

To this end, Ghasemzadeh-Moghaddam et al., analyzing antibody response to 46 staphylococcal antigens in sera of 27 human patients with ST239 MRSA bacteremia, identified a group of highly immunogenic antigens that could be considered when developing a multi-component vaccine or in setting up a passive immunization therapeutic approach [132].

The use of anti-*S. aureus* mAbs in synergy with new-generation antibiotics will, however, continue to be a leading treatment for this aggressive human pathogen.
